# The expression and role of the Lem-D proteins Ankle2, Emerin, Lemd2, and TMPO in triple-negative breast cancer cell growth

**DOI:** 10.3389/fonc.2024.1222698

**Published:** 2024-04-24

**Authors:** Maddison Rose, Joshua T. Burgess, Chee Man Cheong, Mark N. Adams, Parastoo Shahrouzi, Kenneth J. O’Byrne, Derek J. Richard, Emma Bolderson

**Affiliations:** ^1^ Cancer and Ageing Research Program, Centre for Genomics and Personalised Health, School of Biomedical Sciences, Translational Research Institute, Queensland University of Technology, Brisbane, QLD, Australia; ^2^ Department of Medical Genetics, Faculty of Medicine, Institute of Basic Medical Sciences, University of Oslo, Oslo, Norway; ^3^ Cancer Services, Princess Alexandra Hospital, Brisbane, QLD, Australia

**Keywords:** triple negative breast cancer, cancer therapy, Lem-domain proteins, nuclear envelope, inner nuclear membrane

## Abstract

**Background:**

Triple-negative breast cancer (TNBC) is a sub-classification of breast carcinomas, which leads to poor survival outcomes for patients. TNBCs do not possess the hormone receptors that are frequently targeted as a therapeutic in other cancer subtypes and, therefore, chemotherapy remains the standard treatment for TNBC. Nuclear envelope proteins are frequently dysregulated in cancer cells, supporting their potential as novel cancer therapy targets. The Lem-domain (Lem-D) (LAP2, Emerin, MAN1 domain, and Lem-D) proteins are a family of inner nuclear membrane proteins, which share a ~45-residue Lem-D. The Lem-D proteins, including Ankle2, Lemd2, TMPO, and Emerin, have been shown to be associated with many of the hallmarks of cancer. This study aimed to define the association between the Lem-D proteins and TNBC and determine whether these proteins could be promising therapeutic targets.

**Methods:**

GENT2, TCGA, and KM plotter were utilized to investigate the expression and prognostic implications of several Lem-D proteins: Ankle2, TMPO, Emerin, and Lemd2 in publicly available breast cancer patient data. Immunoblotting and immunofluorescent analysis of immortalized non-cancerous breast cells and a panel of TNBC cells were utilized to establish whether protein expression of the Lem-D proteins was significantly altered in TNBC. SiRNA was used to decrease individual Lem-D protein expression, and functional assays, including proliferation assays and apoptosis assays, were conducted.

**Results:**

The Lem-D proteins were generally overexpressed in TNBC patient samples at the mRNA level and showed variable expression at the protein level in TNBC cell lysates. Similarly, protein levels were generally negatively correlated with patient survival outcomes. siRNA-mediated depletion of the individual Lem-D proteins in TNBC cells induced aberrant nuclear morphology, decreased proliferation, and induced cell death. However, minimal effects on nuclear morphology or cell viability were observed following Lem-D depletion in non-cancerous MCF10A cells.

**Conclusion:**

There is evidence to suggest that Ankle2, TMPO, Emerin, and Lemd2 expressions are correlated with breast cancer patient outcomes, but larger patient sample numbers are required to confirm this. siRNA-mediated depletion of these proteins was shown to specifically impair TNBC cell growth, suggesting that the Lem-D proteins may be a specific anti-cancer target.

## 1 Introduction

Triple-negative breast cancers (TNBCs) are a subcategory of breast carcinomas that do not overexpress the human epidermal growth factor receptor 2 (HER2) and lack expression of the estrogen (ER) and progesterone (PR) receptors (1). TNBC is of interest within the scientific community due to the evident discrepancy between survival outcomes for TNBC patients and other breast cancer subtypes.

Specifically, TNBC tumors have been shown to have a significantly earlier and higher rate of recurrence, with recurrence rates peaking at 1–3 years post-therapy (2). The burden of TNBC is emphasized by low 5-year survival rates being 16%, compared with non-TNBC subtypes (3). Despite substantial advances in targeted and personalized cancer therapeutics, chemotherapeutics and surgery remain the primary treatment modalities for TNBC patients due to the lack of hormone receptors on this tumor type (4). Disease reoccurrence, distant metastatic lesions, and acquired resistance are common for TNBC tumors. Therefore, there is evident need for the exploration of novel therapeutic approaches for TNBC to improve patient outcomes.

The nuclear envelope (NE) is a double lipid membrane, originally defined for its role in physically separating the nucleus and cytoplasm within eukaryotic cells (5). This double lipid membrane can be further subcategorized as the inner nuclear membrane (INM) and outer nuclear membrane. There is substantial literature demonstrating that the INM proteins are required to maintain cellular functioning. Dysregulation or mutations of these proteins have been shown to be involved in disease pathophysiology, including cancer and progeria syndromes (4, 6–8).

The INM is host to several proteins, including the Lem (LAP2, Emerin, MAN1 domain, and Lem-D) domain proteins, which share a ~45-residue Lem-domain (Lem-D) that binds to and interacts with the INM protein, Banf1 (9–12). The Lem-D proteins can be further categorized into numerical groups (Groups I–III) based on their membrane topology. Group I proteins, Emerin, Lap2β, Lemd1, and Lap2α, possess nucleoplasmic domains, and a transmembrane domain, except for Lap2α (10–12). Group II proteins, MAN1 and Lemd2, have two transmembrane domains and a winged helix DNA-binding MSC domain (9, 13, 14). Finally, Group III proteins are functionally diverse from the other sub-groups, Ankle2 includes an endoplasmic reticulum transmembrane domain and Ankle1 undergoes nucleo-cytoplasmic shuttling (15, 16). Given the Lem-D proteins have broad membrane topology, it is conceivable that this translates to diverse roles in tumorigenesis (13). Collectively, these Lem-D proteins have been shown to participate in each of the hallmarks of cancer: aberrant cell cycle progression, cell migration and invasion, aberrant mitosis, dysregulated DNA repair mechanisms, upregulated proliferation, and dysregulated cell signaling (14–17). Expression of several Lem-D proteins is known to be altered in numerous cancer models, including breast cancer, further supporting a role of the Lem-D proteins in TNBC tumorigenesis (17–20).

Here, we investigate the role of several Lem-D proteins, Ankle2, Emerin, TMPO, and Lemd2, in TNBC tumorigenesis, establishing that depletion of several INM proteins has anti-proliferative effects and induces apoptosis on TNBC cells, supporting the assertion that targeting the Lem-D proteins may be an efficacious strategy to treat TNBC.

## 2 Methodology and materials

### 2.1 Reagents

All chemical reagents were purchased from Sigma-Aldrich (Sigma-Aldrich, Saint Louis, MO, USA), unless otherwise stated.

### 2.2 Antibodies

Antibodies used were as follows: anti-Emerin (5430, Cell Signaling Technology, Danvers, MA, USA 1:500 for IF, 1:1000 for IB), anti-Lemd2 (PA553589, Thermo Fisher Scientific, Waltham MA, USA 1:300 for IF, 1:1000 for IB), anti-Ankle2 (GTX120698, Genetex, Irvine, CA, United States 1:200 for IF, 1:1000 for IB), and anti-TMPO (L3414-.2ML, Sigma-Aldrich, Saint Louis, MO 1:500 for IF, 1:1000 for IB), anti-GAPDH (glyceraldehyde-3-phosphate dehydrogenase) (D16H11, Cell Signaling Technology, 1:4000 for IB), and anti–Gamma-Tubulin (T6557, Sigma-Aldrich, 1:3000 for IB). Fluorescent secondary antibodies used were Alexa Fluor 488 (Cat# A32766, Molecular Probes, Thermo Fisher Scientific 1:200 for IF) and 594 (Cat# A32754, Molecular Probes, Thermo Fisher Scientific, 1:200 for IF), IRDye^®^ 800CW Donkey anti-Mouse IgG Secondary Antibody (926-32212, LiCor Bioscience, Lincoln, NE, USA), and IRDye^®^ 680RD Donkey anti-Rabbit IgG Secondary Antibody (926-68073, LiCor Bioscience).

### 2.3 Cell culture

BT549, Hs578T, MDA-MB-231, and MDA-MB-468 cells were utilized as representative TNBC cells. MCF10A cells were used as a non-malignant, breast tissue–derived control. BT549 and Hs578T cells were cultured in RPMI (Thermo Fisher Scientific), and MDA-MB-231 and MDA-MB-468 cells were cultured in DMEM (Thermo Fisher Scientific). All cell lines were obtained from the American Type Culture Collection (ATCC) (Manassas, VA, USA). All cell lines were supplemented with 10% fetal bovine serum (FBS) (Thermo Fisher Scientific). MCF10A cells were maintained in DMEM/F12, supplemented with 20% FBS, 100 ng/mL Cholera Toxin (Sigma-Aldrich) 20ng/mL EGF and 0.01 mg/mL Insulin (Sigma-Aldrich). All cells were cultured at 37°C in an atmosphere of 5% CO_2_.

### 2.4 siRNA transfections

Control (4390843) and INM silencer select siRNAs [Ankle2 (s23124), TMPO (s24159), Emerin (s2245840), and Lemd2 (s48070) siRNAs] were purchased from Thermo Fisher Scientific. RNAiMax (Invitrogen, Waltham, MA, USA) was used to transfect siRNA, as per manufacturer guidelines.

### 2.5 Immunoblotting

Cells were lysed (lysis buffer: 20 mM HEPES pH 7.5, 250 mM KCl, 5% glycerol, 10 mM MgCl2, 0.5% Triton X-100, protease/phosphatase inhibitor cocktail (Thermo Fisher Scientific), sonicated and cleared by centrifugation. Fifteen microgram of protein was separated on a 4%–12% BIS-TRIS gel (Invitrogen) prior transfer to nitrocellulose membrane. Following transfer the membrane was blocked in Intercept Blocking Buffer (LiCor Bioscience) for 30 min at room temperature. Immunoblotting was carried out with the indicated antibodies (see above for antibody details), incubated with the indicated primary antibodies for 1h at room temperature in phosphate-buffered saline solution (PBS-T), washed 3 times in PBS-T, prior to 1h room temperature incubation in Alexa-conjugated secondary antibodies in PBS-T and washed 3 times in PBS-T at room temperature. Anti-GAPDH or γ-tubulin antibodies were used as a loading control. Immunoblots were imaged using an Odyssey infrared imaging system (LiCor Bioscience).

### 2.6 Immunofluorescent microscopy

Immunofluorescence was performed as previously (21). Briefly, 5,000 cells/well were seeded in a 96-well plate and allowed to adhere for 24h. Cells were pre-treated with extraction buffer for 5 min to visualize chromatin bound protein, prior to fixation in 4% paraformaldehyde for 20 min at room temperature. Cells were permeabilized for 5 min in 0.2% Triton X-100/PBS prior to blocking for 30 min in 3% bovine serum albumin/PBS. Subsequently, cells were incubated in indicated primary antibodies for 1h at room temperature in PBS, washed 3 times in PBS, prior to 1h room temperature incubation in Alexa-conjugated secondary antibodies in PBS. Cells were then counter-stained in Hoechst 33342 in PBS (1μg/mL) for 5 min at room temperature, washed 3 times in PBS and imaged on a DeltaVision pDV deconvolution microscope with 100×/1.42 oil objective (Applied Precision Inc, Issaquah, WA, USA). ImageJ was utilized to assemble images. High-throughput imaging was performed using the IN Cell Analyzer 6500 Imaging System (GE HealthCare Life Sciences, Arlington Heights, IL, USA). Nuclear, cytoplasmic, and cellular staining intensity was analyzed using the IN Cell Investigator software (GE HealthCare Life Sciences) with a minimum of 200 nuclei quantified/per condition.

### 2.7 Nuclear envelope localization and morphology quantification

Immunofluorescent staining and imaging were conducted as above. Localization and quantification were performed as previously described (12). Briefly, a minimum of 200 cells/condition were manually determined to have Lem-D proteins localized/not localized to the NE and for their “nuclear roundness” using the nuclear form factor function (form factor = $\frac{4\times \text{π}\times area}{perimeter^{2}}$ ) within the IN Cell Investigator software (GE HealthCare Life Sciences) suite. Nuclear form factor is also defined as the measure of nuclear circularity or as the nuclear contour ratio; this measurement was deemed the most suitable measurement of nuclear circularity as existing literature demonstrates that it reflects the extent of abnormality in multi-lobed nuclei more accurately than other measurements, including solidity or eccentricity (22, 23). As a secondary technique, nuclear roundness was assayed by manually determining cells to have normal/abnormal nuclear morphology.

### 2.8 Proliferation assay

Seventy-two hours following transfection, 500/cells per well were seeded at sub-confluence into a 96-well plate and allowed to adhere for 24h. Following adhesion, the 96-well plate was placed into an Incucyte S3 Live Cell Imaging System (Essen Bioscience, Ann Arbor, MI, USA) and an unlabeled cellular confluence assay was utilized to determine proliferation rate over a 5-day period. Proliferation curves are representative of results and area under the curve (AUC) graphs represent the mean and S.D. of three independent experiments.

### 2.9 Apoptosis assay

Cell death was quantified using an Annexin V-FITC apoptosis kit (ALX-850-020-KI02, Enzo Life Sciences, Farmingdale, NY, USA). Five days post-transfection, cells were enzymatically lifted and media containing floating cells was collected. Cells were then resuspended at 1 × 10^6^ cells/mL in 488-conjugated anti-annexin (1:40), containing binding buffer. Cells were incubated for 20 min at room temperature and stained with propidium iodide (1 mg/mL). Cells were assayed using a CytoFLEX Flow Cytometer (Beckman Coulter Life Sciences, Indianapolis, IN, USA), and data were analyzed using FlowJo analysis software.

### 2.10 Bioinformatics and statistical analysis

Data from the GENT2 database (http://gent2.appex.kr/gent2/) were used to assess Lem-D protein transcript levels across breast cancer stages and histologies compared to surrounding healthy tissue (24). Box plots show median expression levels for each gene of interest with interquartile ranges and notches show the 95% confidence intervals. Significance levels were determined by unpaired Mann–Whitney U tests. Data extracted from the GENT2 database included breast cancer and non-cancerous breast tissues from 72 publicly available datasets (Ankle2: 4293 cancer samples and 92 non-cancerous samples).

Plot functions within the cBioPortal for Cancer Genomics (https://www.cbioportal.org/) were utilized to analyze potential correlations between mRNA expression of genes of interest (*n* = 312, four Grade I, 38 Grade II, and 270 Grade III samples) based on TNBC tumor grade. Raw TCGA data were obtained via the cBioPortal for Cancer Genomics and compiled in Graph Pad Prism 9.0 and one-way analyses of variance (ANOVAs) were utilized to establish statistical significance.

Kaplan–Meier Plotter database (http://kmplot.com/analysis/index.php?p=service) was used to perform survival analysis for all breast cancer samples based on Lem-D protein mRNA and protein expression levels as described (25). Expression was categorized as high- or low-expressed based on the median mRNA expression within the database. For mRNA analysis, sample sizes were as follows: Lemd2 (Geneprobe set – 2224980, *n* = 943, low expression = 470, high expression = 473), TMPO (Geneprobe set – 203432, *n* = 1879, low expression = 940, high expression = 939), Ankle2, (Geneprobe set – 212200, *n* = 1879, low expression = 947, high expression = 932), and Emerin (Geneprobe set – 209477, *n* = 1887, low expression = 944, high expression = 935). For protein analysis, the Tang dataset was utilized to analyze the correlation between Lemd2 and TMPO expression and OS (*n* = 126), and the Liu dataset was used to analyze the correlation between Ankle2 and Emerin expression and OS (*n* = 65) (26, 27). A log-rank test and Cox proportional hazard analysis were used to determine the statistical significance of survival outcomes.

Unless otherwise stated, data are presented as mean values and error bars represent SD from three biologically independent experiments. Normality was assessed using the Shapiro–Wilk test in Graph Pad Prism. If normality test was passed, statistical analysis was performed using a two-tailed Student’s t-test or one-way ANOVAs. If normality test failed, statistical analysis was performed using a Mann–Whitney U test or Kruskal–Wallis H test.

## 3 Results

### 3.1 Expression of the Lem-domain proteins in patient samples

**Figure 1:**
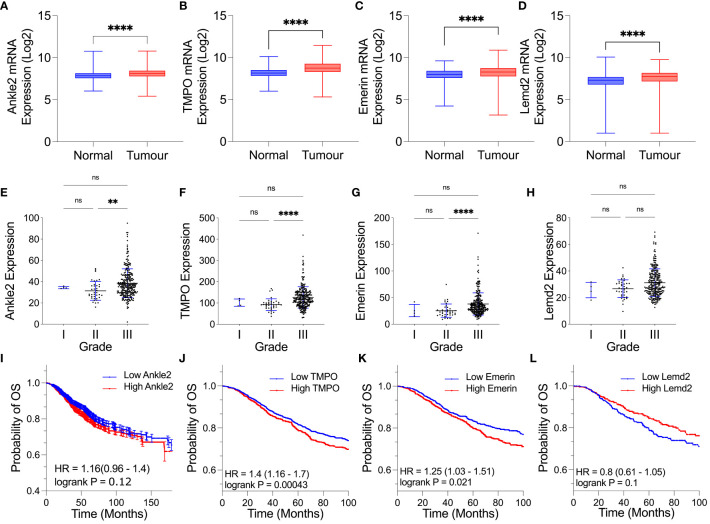
Expression of several Lem-D proteins is significantly elevated in TNBC tumor patient samples. **(A–D)** Box plots of Lem-D gene expression comparing normal and breast tumor patient samples utilizing the Gene Expression database of Normal and Tumor tissues 2 (GENT2) database: **(A)** Ankle2 expression, **(B)** TMPO expression, **(C)** Emerin expression, and **(D)** Lemd2 expression. **(E–H)** Graphical representation of expression of the Lem proteins in Stages I, II, and III TNBC patient samples utilizing The Cancer Genetics Atlas (TCGA) database: **(E)** Ankle2 **(F)** TMPO **(G)** Emerin, and **(H)** Lemd2 expressions. **(I–L)** Kaplan–Meier values for Lem-D gene expression in breast cancer patients showed that high mRNA expression of Lem-D genes was associated with a decrease in the probability of patient overall survival. Lem-D mRNA transcript expression was categorized as high or low expressed based on the median mRNA expression within the database. The effect of **(I)** Ankle2, **(J)** TMPO, **(K)** Emerin, and **(L)** Lemd2 mRNA expression on patient overall survival. For **(A–H)**: error bars denote standard deviation of the mean. Statistical significance was calculated using a Kruskal–Wallis test: ****p< 0.0001, **p< 0.0021. For **(I–L)**: statistical significance was calculated using a log-rank *p*-test. ns, not significant.

To establish whether the expression of the Lem-D proteins was dysregulated in breast cancer, the mRNA fold change of each Lem-D protein was analyzed using the GENT2 dataset (**Figures 1A–D**) (24). *Ankle2*, *TMPO*, *Emerin*, and *Lemd2* were significantly overexpressed in breast tumor samples, in comparison to adjacent normal tissue. However, for most of the transcripts investigated, the difference in expression was only a 0.1- to 0.3-fold change (**Figures 1A–D**). Furthermore, expression data for Grades I–III TNBC samples within the TCGA dataset were utilized to establish correlations between tumor stage and expression of Lem-D transcripts (**Figures 1E–H**). The only datasets that reached statistical significance were the difference in mRNA between Grades II and III TNBC carcinomas. No statistically significant difference was observed between Grades I and II or I and III tumors. Although, it should be noted that the datasets contain less than 10 samples, which could impact the overall significance of the results. Given this dataset did not include values for non-cancerous samples, we were unable to establish the difference in expression of the Lem-D proteins in each grade and non-cancerous tissue.

Kaplan–Meier plots were generated to investigate the level of correlation between Lem-D protein mRNA expression and overall survival (OS), defined as the time from diagnosis to death, in breast cancer patients. Plots were generated utilizing the mRNA genechip data available within the KM plotter online database (**Figures 1I–L**). Kaplan–Meier survival probabilities are generated at each datapoint based on the number of surviving patients, relative to the number of patients at risk. *TMPO* mRNA expression was negatively correlated with breast cancer patient OS (**Figure 1J**). *Ankle2* and *EMD* mRNA expressions were also negatively correlated with OS, however, were not statistically significance (**Figures 1I, K**). In contrast, *Lemd2* expression positively correlated with OS to a level that did not reach statistical significance (**Figure 1L**). Kaplan–Meier plots were also generated for protein-expression datasets; however, datasets available were of a small size (*n* = 65 for Ankle2 and Emerin and *n* = 126 for TMPO and Lemd2). From these datasets, protein expression of the Lem-D proteins showed a negative relationship with OS, reaching statistical significance for Ankle2, EMD, and Lemd2. However, statistical significance was not reached for the correlation between protein expression of TMPO and breast cancer patient OS (**Supplementary Figure S1**). Together, these analyses suggest that overexpression of the Lem-D proteins is generally associated with lower overall patient survival.

### 3.2 Expression and localization of the Lem-D proteins in TNBC cells

Given the Lem-D transcripts, *Ankle2*, *TMPO*, *Emerin*, and *Lemd2*, were overexpressed in breast cancer patient samples and overexpression largely associated with poorer patient outcomes, we next investigated whether the Lem-D proteins were similarly overexpressed in TNBC cell lines.

**Figure 2:**
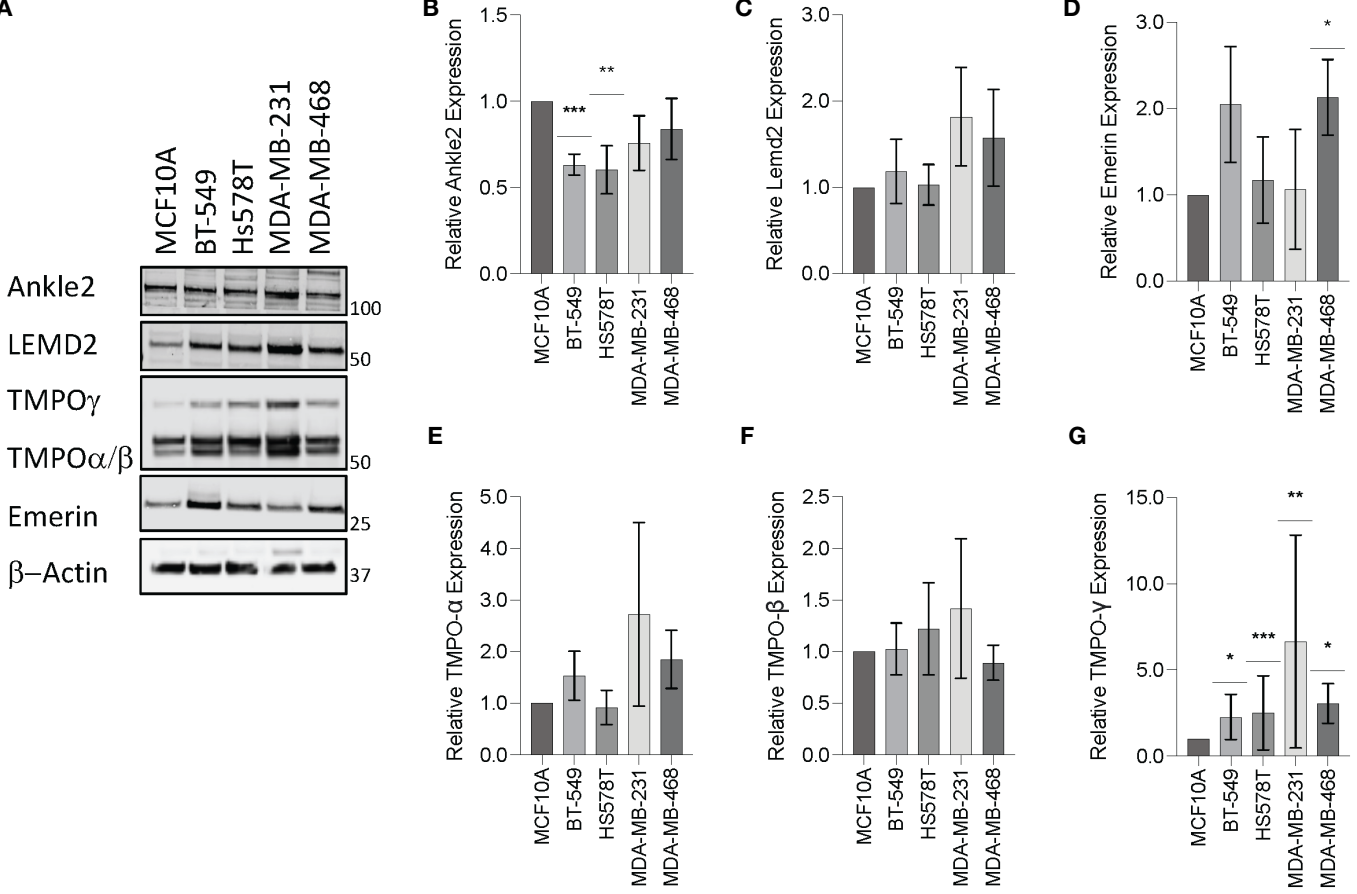
Expression of the Lem-domain proteins in TNBC cells. **(A)** Representative Western blot of BT549, Hs578T, MDA-MB-231, and MDA-MB-468 whole cell lysates showing expression of Lemd2, Ankle2, Emerin, and TMPO isoforms in TNBC cell lines, in comparison to the control MCF10A non-cancerous breast tissue cells. β−Actin was utilized as a loading control to allow for standardization via densitometry in ImageJ Software **(B–G)** Graphs represent densitometry analysis of Lem protein expression determined via Western blot analysis in TNBC normalized to non-cancerous MCF10A cells: **(B)** Ankle2 **(C)** Lemd2 **(D)** Emerin, and **(E–G)** TMPOα, β, and γ. Graphed values represent results from three individual repeats and error bars denote standard deviation of the mean. Statistical significance was calculated using an unpaired t-test: ***p< 0.0002, **p< 0.0021, *p< 0.0332.

To investigate the expression of the Lem-D proteins in TNBC, immunoblotting was conducted in the representative TNBC cell lines: BT549, Hs578T, MDA-MB-231, and MDA-MB-468, in comparison to epithelial MCF10A cells as a non-cancerous breast tissue cell line. Ankle2 expression was significantly downregulated in the BT549 and Hs578T TNBC cell lines, in comparison to the non-cancerous MCF10A cells (**Figures 2A, B**). In contrast, Emerin was shown to be over-expressed in one out of four of the TNBC cells, in comparison to MCF10A cells, whereas there were no significant changes in LEMD2 protein levels (**Figures 2A, C, D**). There are three main isoforms of TMPO, and these were all detected by the TMPO antibody used in this study. While there were no significant changes in TMPOα or β protein levels, TMPOγ was shown to be significantly increased in all four TNBC cell lines tested (**Figures 2A, E–G**).

**Figure 3:**
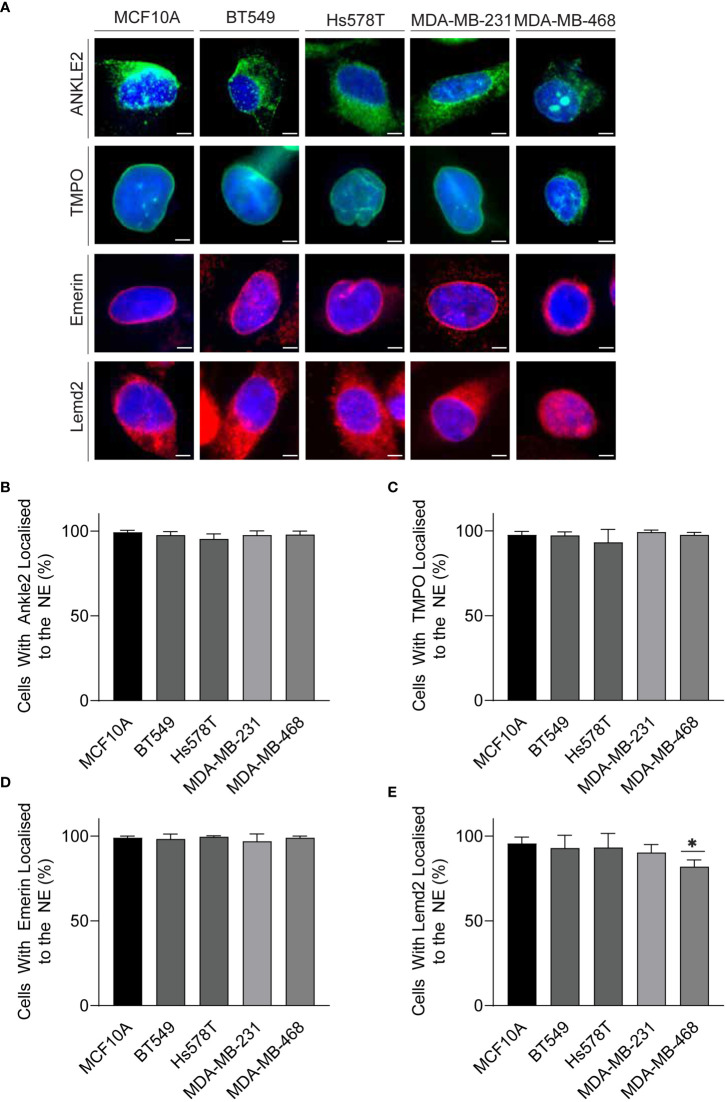
The Lem-D proteins are consistently localized in TNBC cells. **(A)** Representative immunofluorescent microscopy images of MCF10A, BT549, Hs578T, MDA-MB-231, and MDA-MB-468 cells. Cells were stained with Ankle2, TMPO, Emerin, and Lemd2 antibodies. Cells were counterstained with Hoechst 33342 (blue). **(B–E)** Quantification of the portion of cells with individual Lem-D proteins localized to the NE. **(B)** Ankle2, **(C)** TMPO, **(D)** Emerin, and **(E)** Lemd2. Quantifications are based on 200 cells/condition in at least three experimentally independent experiments. Error bars denote standard deviation of the mean. Scale bars = 10 µM. Statistical significance was calculated using an unpaired t-test: *p< 0.0332.

Mislocalization of proteins is a well-recognized characteristic of tumor cells (28). Therefore, we next investigated whether localization of the Lem-D proteins was maintained in TNBC cells. Immunofluorescent microscopy of TNBC cells demonstrated that localization of the Lem-D proteins was largely consistent with that of non-cancerous MCF10A cells (**Figure 3**; **Supplementary Figure S2**). Across all tumorigenic cell lines, Emerin and TMPO staining maintained clear NE localization, with some nuclear staining, in TNBC and MCF10A cells. Ankle2 staining was predominately NE localized, with minor cytoplasmic staining, likely attributed to endoplasmic reticulum localized Ankle2 (29). Similarly, Lemd2 predominately localized to the NE, with evident cytoplasmic staining in TNBC and MCF10A cells. There was minimal variation between the percent of TNBC and MCF10A cells where the Lem-D proteins did not localize to the NE (**Figures 3B–E**). However, in MDA-MB-468 cells there was a small but significant decrease in the percent of NE localized Lemd2 cells, compared to MCF10As (**Figure 3E**).

**Figure 4:**
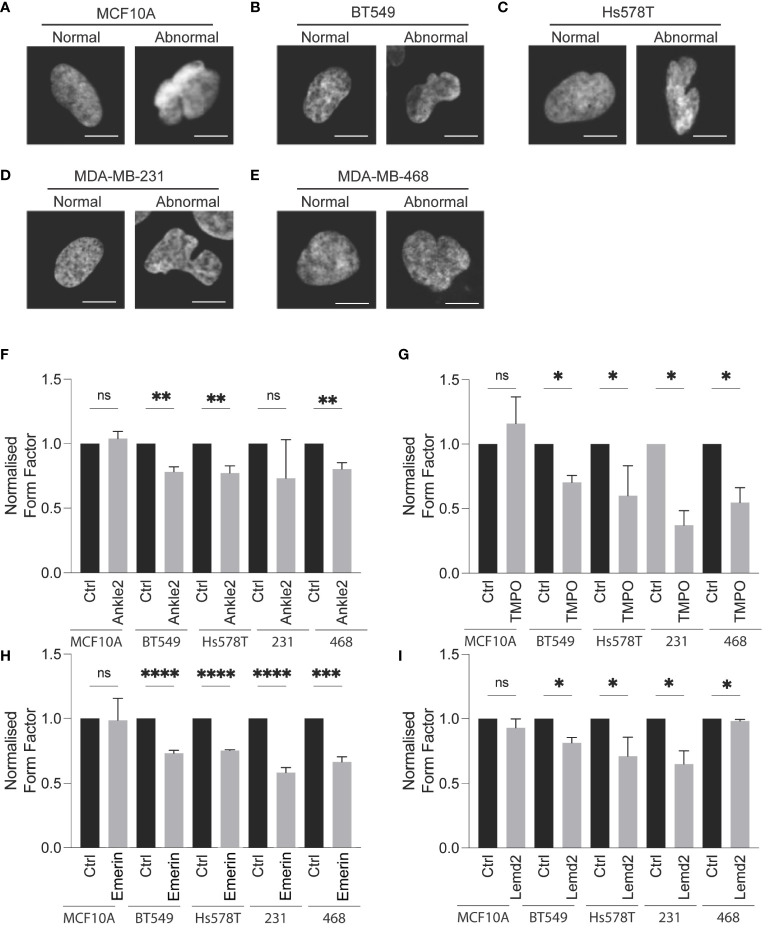
siRNA-mediated depletion of the Lem-D proteins induces aberrant NE morphology in TNBC cells. Representative immunofluorescent microscopy images of MCF10A and TNBC cells transfected with control and Lem-domain (Lem-D) protein siRNAs. Cells were stained with Hoechst 33342 to visualize the nucleus. Representative cells categorized to have normal and abnormal nuclear morphology: **(A)** MCF10A **(B)** BT549 **(C)** Hs578T **(D)** MDA-MB-231, and **(E)** MDA-MB-468. Quantification of cells with aberrant nuclear morphology in Control and Lem-D protein siRNA transfected cells. Normalized nuclear form factor values for: **(F)** Ankle2, **(G)** TMPO, **(H)** Emerin, and **(I)** Lemd2 siRNA. Form Factor score of 1 = perfect round nucleus. Quantifications are based on 200 cells/condition in at least three independent experiments. Error bars denote standard deviation of the mean. Statistical significance was calculated using an unpaired t-test: ****p< 0.0001, ***p< 0.0002, **p< 0.0021, *p< 0.0332. ns, not significant.

Atypical nuclear morphology is a hallmark of cancer cells and aberration of NE proteins induces abnormal nuclear morphology in cells (7, 30–32). Therefore, we sought to investigate whether Lem-D protein depletion induces abnormal nuclear morphology. Protein expression of Ankle2, Emerin, TMPO, and Lemd2 was depleted via siRNA and confirmed by immunofluorescent microscopy [**Supplementary Figure S3** (168h post-transfection), **Supplementary Figure S4** (72h post-transfection)]. Nuclear morphology was analyzed via immunofluorescent microscopy and quantified using the form factor analysis of InCarta software and values were verified by visually categorizing nuclei as having abnormal/normal morphology (**Figure 4**; **Supplementary Figure S5**). siRNA-mediated depletion of all Lem-D proteins significantly decreased the form factor value at 96h post-transfection of TNBC cells, in comparison to control siRNAs. Furthermore, Lem-D protein depletion did not significantly alter form factor values from respective controls in MCF10A cells (**Figure 4**). Visual quantification of nuclei as having abnormal/normal morphology supported these findings, demonstrating that siRNA-mediated depletion of Lem-D proteins significantly increased the percent of TNBC cells with abnormal nuclear morphology, but not MCF10A cells (**Figure 4**; **Supplementary Figure S5**). Collectively, suggesting a tumor-specific role for the Lem-D in maintaining nuclear morphology.

### 3.3 Depletion of the Lem-domain proteins inhibits TNBC cell growth

To investigate the role of the Lem-D proteins in TNBC growth, an Incucyte S3 direct cell count proliferation assay was conducted to establish changes in proliferative capacity of TNBC and MCF10A cells following siRNA-mediated depletion of the Lem-D proteins. BT549 and MDA-MB-231 were selected as representative TNBC cell lines.

**Figure 5:**
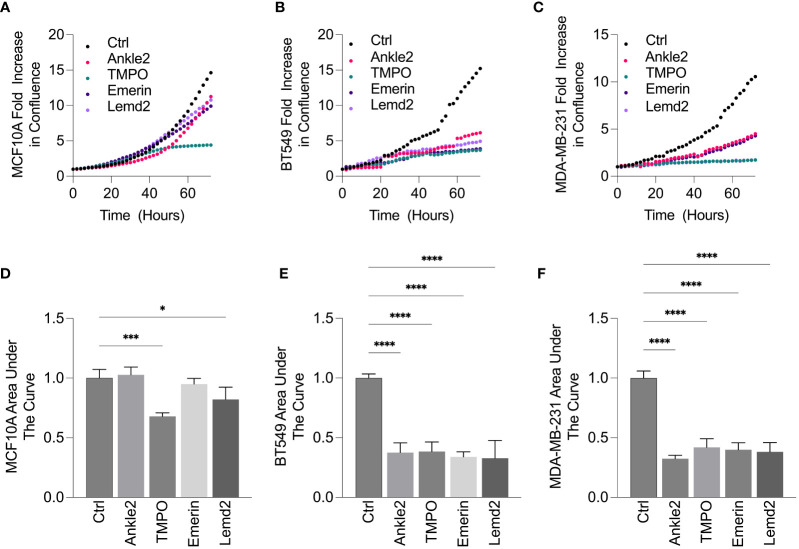
siRNA-mediated depletion of Lem-D proteins inhibits TNBC cellular proliferation. **(A–E)**. Representative proliferation curves from 72h post-transfection with Control and individual Lem-D protein siRNAs using the Incucyte S3 live cell imaging system in MCF10A and TNBC cells. **(A)** MCF10A **(B)** BT549 and **(C)** MDA-MB-231. **(D–F)** Relative area under the proliferation curve for **(A–C)** from at least three independent experiments. Error bars denote standard deviation of the mean. Values are normalized back to Control siRNA for respective cell lines. Statistical significance was calculated using an unpaired t-test: ****p< 0.0001, ***p< 0.0002, *p< 0.0332.

Ankle2 and Emerin depletion did not significantly impact upon the proliferation of non-cancerous MCF10A cells, as demonstrated by the proliferation graphs and AUC analysis (**Figures 5A, D**). In contrast, TMPO and Lemd2 depletion had a 20%–30% reduction in MCF10A cell proliferation, as demonstrated via AUC analysis compared to the cells transfected with control siRNA (**Figures 5A, D**). Lemd2 depletion was shown to decrease the proliferative capacity of the tumorigenic BT549 and MDA-MB-231 cells by ~50%–65%. This suggests some level of tumor specificity, as the anti-proliferative effect of Lemd2 depletion in TNBC cells was twofold to 2.5-fold higher than observed in MCF10A cells. (**Figures 5B–F**). The marked antiproliferative effect following TMPO depletion in BT549 and MDA-MB-231 cells was similar in MCF10A cells, suggesting that siRNA-mediated depletion of TMPO is not likely to be a targeted mechanism of inhibiting TNBC cell growth. Finally, Ankle2 and Emerin depletion showed similar anti-proliferative outcomes in BT549 and MDA-MB-231 cells, reducing the proliferation rate to ~50% of respective endogenous rates (**Figures 5B–F**).

**Figure 6:**
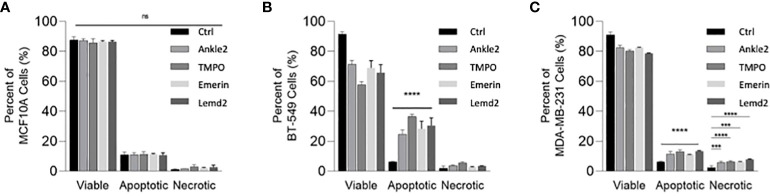
siRNA-mediated depletion of the Lem-D proteins induces TNBC cell death. Graphs represent percent of live, apoptotic (early and late apoptotic) and necrotic cells 5 days post-transfection with Control, Ankle2, TMPO, Emerin, and Lemd2 siRNAs **(A)** MCF10A **(B)** BT549 **(C)** MDA-MB-231 cells. Graphed values represent results from three individual repeats and error bars denote standard deviation of the mean. Statistical significance was calculated using an unpaired *t*-test: ****p< 0.0001, ***p< 0.0002. ns, not significant.

Given that Lem-D protein depletion was shown to inhibit tumor cell proliferation, we next investigated whether this was due to cell death. An Annexin V/PI apoptosis assay was conducted 5 days post-transfection with the Lem-D and control siRNAs and measured by flow cytometry (**Figures 6A–C**; **Supplementary Figure S6**). In MCF10A cells, transfection with the Lem-D siRNAs did not significantly increase cell death, compared to control siRNA (**Figure 6A**). However, depletion of all Lem-D proteins significantly increased the percentage of early or late-apoptotic cells in the BT549 cell line, in comparison to the control siRNA (**Figure 6B**). Similarly, Lem-D protein depletion in MDA-MB-231 cells significantly increased the percent of apoptotic or necrotic cells, relative to the control (**Figure 6C**).

## 4 Discussion

The Lem-D proteins have been shown to be dysregulated in various cancer models and, suppression or silencing of several of these proteins is known to produce anti-proliferative effects in breast, colon, lung, gastric, and cervical cancer models (14, 17, 33–36). Our findings build upon the existing knowledge of the roles of Lem-D proteins in tumor cells and demonstrate the role of several Lem-D proteins: Ankle2, TMPO, Emerin, and Lemd2 in TNBC growth and cell survival. This study provides novel insight into the capacity of this protein family as potentially exploitable as an anti-cancer therapy.

### 4.1 Ankle2 in triple-negative breast cancer

Ankle2 is a Lem-D protein required for maintaining NE structural integrity and has a multifaceted role in post-mitotic nuclear reassembly. Specifically, Ankle2 is essential for preserving appropriate dephosphorylation of Banf1 during mitosis, via both inhibiting the Banf1 phosphorylating kinase, VRK1, and upregulating PP2A-mediated dephosphorylation, to promote chromatin recruitment (37). To understand the role of Ankle2, and the other Lem-D proteins, in breast cancer tumorigenesis, bioinformatic analysis of the GENT2 database was conducted to establish whether mRNA levels of the Lem-D proteins were altered in breast cancer samples, in comparison to non-cancerous tissue adjacent to tumor margins (24). We demonstrated that *Ankle2* transcripts were overexpressed in breast cancer samples, compared to non-cancerous tissue, and the extent of overexpression negatively correlated with breast cancer patient OS.

Despite this initially positive trend, we observed significant downregulation of Ankle2 expression in half of the TNBC cells compared to a non-cancerous control. In contrast to our findings, Ankle2 overexpression has previously been reported in ER-positive breast cancer models, with Ankle2 being identified to have an essential role in promoting ERα DNA-binding and transactivation activity (17). Given that TNBC cells lack the ER receptor, it is conceivable that this may contribute to the lack of Ankle2 overexpression observed in TNBC cells specifically.

Consistent with earlier findings in both mammalian and Drosophila models, we observed Ankle2 localization to the NE and ER in TNBC cells (29). Furthermore, we demonstrated that siRNA-mediated depletion of Ankle2 induced a markedly abnormal nuclear phenotype in TNBC cells, consistent with prior findings in U-2OS osteosarcoma cells and HeLA cervical carcinoma cells (38, 39). Ankle2’s role in maintaining nuclear integrity has been previously attributed to the role of Ankle2’s phosphorylation status in NE breakdown and reformation (37, 38). Consistent with our observations, Ankle2 depletion by CRISPR/Cas9 technology has been previously shown to impair the mechanical stability of the nucleus and induce chromosomal instability via disrupting the association between Banf1, Lamin A, and Lap2a with the chromosomes following mitosis (39). LEM-4L silencing has been previously shown to disrupt Banf1 dephosphorylation and subsequent post-mitotic NE reformation in *C. elegans* (37). Together, these findings suggest that the abnormal nuclear phenotype observed in TNBC cells following Ankle2 depletion may arise due to ineffective post-mitotic NE reformation. Furthermore, our data demonstrates that siRNA-mediated Ankle2 depletion inhibits cell proliferation and induces cell death in a largely tumor-specific manner. While our investigation is the first to propose a role for Ankle2 TNBC proliferation, previous studies in hormone-positive breast cancer cells and HeLa cells support this observation (17, 39).

Based on the known cellular functions of Ankle2, it can be hypothesized that Ankle2 depletion may impair TNBC proliferation and induce cell death due to the inability to reform the NE following mitosis in the highly mitotic TNBC cells, resulting in mechanically vulnerable cells and potential cell death.

### 4.2 Emerin in triple-negative breast cancer

Emerin is an inner NE protein known to be involved in maintaining the nuclear morphology of interphase cells and post-mitotic nuclear reformation. We demonstrate *EMD* transcript overexpression in breast cancer patient samples. EMD protein was also increased in two out of four TNBC cell lysates tested, when compared to non-cancerous cell lines. However, previous investigations have produced conflicting results regarding the expression of Emerin protein in breast cancer samples, with one study showing a decrease in Emerin protein levels (40) and one showing an increase in comparison to human primary breast epithelial cells (20). Our findings also demonstrated that *EMD* expression was shown to negatively correlate with patient outcomes suggesting a possible role for Emerin in breast cancer cell growth and proliferation.

Consistent with prior findings in both mammalian cells and *C. elegans*, we observed Emerin localization to the NE in both the MCF10A and TNBC cells (41, 42). Like our Ankle2-related findings, we observed that siRNA-mediated depletion of Emerin induced an abnormal nuclear morphology in TNBC cells. Previous studies have shown that deletion of regions of the *EMD* sequence has been shown to induce improper centromere and tubulin network localization and increase mitotic time (36, 43). Therefore, this suggests that abnormal nuclear morphology may arise following Emerin depletion due to failed or defective mitotic events. The role of Emerin in maintaining appropriate mitotic progression may also contribute to the anti-proliferative effect and cell death observed following Emerin depletion in TNBC cells. Numerous prior investigations have reported a role for Emerin in cellular proliferation, and that siRNA-mediated depletion of Emerin inhibited cellular proliferation through multiple proposed methods (44, 45). However, it has been previously shown that Emerin directly binds beta-catenin, a signaling factor of the Wnt pathway which is commonly dysregulated in tumor cells. Specifically, Emerin null cells have been shown to have suppressed beta-catenin activity and upregulated cellular proliferation (46). GFP-Emerin overexpression has also been shown to decrease tumor size in mice models (47). Collectively, our findings and prior literature indicate that Emerin is likely to have a multifaced role in tumor cell growth and proliferation, which may be impacted by several underlying factors, therefore, indicating the need for further investigation into the underlying role of Emerin in tumor cell growth.

### 4.3 Lemd2 in triple-negative breast cancer

Lemd2 is an INM protein with a known role in several cellular processes, including nuclear organization. Cellular investigations in HeLa cells and *C. elegans* also suggest a role of Lemd2 in maintaining nuclear morphology due to its interactions with chromatin and the nuclear lamina, with siRNA-mediated depletion of Lemd2 inducing a similar nuclear phenotype to that observed within our own investigations (48, 49).

Unlike the other Lem-D proteins investigation, Lemd2 expression was shown to positively correlate with OS in breast cancer patients. While our study is the first to investigate Lemd2 expression in breast cancer, a previous study has shown Lemd2 overexpression in prostate adenocarcinoma models (16). Furthermore, our findings demonstrated the localization of Lemd2 to the NE and cytosol, which we hypothesized to be lysosomal. This NE localization of Lemd2 is consistent with prior findings in *S. pombe* yeast and multiple human cell lines, including U-2OS and HeLa cells (32, 50). While our investigation was the first to propose the lysosomal localization of Lemd2, Lemd2 has been shown to interact with ESCRT-III, a key protein involved in maintaining the lysosomal membrane (50). Similarly, WT Lemd2 overexpression has been shown to have similar localization in U-2OS cells (51). However, further investigations are required to validate the proposed localization of Lemd2 to the lysosomes.

Additionally, Lemd2 depletion was shown to induce an abnormal nuclear phenotype in the TNBC cell lines but not non-cancerous MCF10A cells. Given that Lemd2 has been previously identified to have essential roles in NE reformation during mitosis, it can be proposed that TNBC cells may exhibit abnormal nuclear morphology following improper NE reformation during mitosis (50).

Our findings also demonstrated that siRNA-mediated depletion of Lemd2 significantly impaired cellular proliferation and induced apoptosis in a TNBC cell–specific manner. While the exact tumor inhibiting mechanism is yet to be established, Lemd2’s role in inducing aberrant nuclear morphology and inhibiting the growth of TNBC could be attributed to Lemd2’s role in NE rupture repair. Lemd2 is required for the recruitment of ESCRT-III mediated repair machinery to the site of NE ruptures (52). Therefore, given abnormal nuclear morphology and uncontrollable proliferation are pre-existing hall marks of tumor cells, it is conceivable that these collectively result in NE ruptures which are unable to be repaired in Lemd2-deficient cells. Therefore, further inducing aberrant nuclear morphology, inhibiting tumor cell growth, and inducing cell death. However, Lemd2 has also been shown to have multiple other cellular functions, including participation within the MAPK signaling pathway. Therefore, potential change in the activity of these pathways should also be investigated to further elucidate Lemd2’s role in tumor cell growth (53).

### 4.4 TMPO in triple-negative breast cancer

TMPO is alternatively spliced to produce three main isoforms, several of which have been linked to nuclear mechanics and are known to be NE localized (54). The TMPO isoform, TMPOα, has been indicated to have a role in maintaining nuclear organization via stabilizing higher order chromatin organization (55). TMPOβ is also known to participate in nuclear growth in Xenopus *laevis* models, with the treatment of cells with the human TMPOβ fragment 1–187 inducing a dose-dependent formation of scalloped nuclei phenotype, consistent with that observed within our investigations (54). Our data indicate that TMPO may be overexpressed in breast cancer tumors, as demonstrated by patient data and that the TMPOγ isoform protein is significantly increased in TNBC cell lines. Previous investigations have demonstrated TMPO overexpression in multiple tumor cell lines, including breast, colorectal, cervical, and pancreatic cancer models (35, 56–59). Furthermore, we demonstrate that TMPO overexpression may play a role in breast cancer tumor growth pathways, given that mRNA expression was negatively correlated with patient outcomes.

Consistent with earlier findings, we observed compelling NE localization of TMPO (60). Furthermore, siRNA-mediated TMPO depletion was shown to induce aberrant nuclei morphology in TNBC cell lines but not non-cancerous MCF10A cells. Given the recognized cellular functions of TMPO, it can be proposed that TMPO-depleted TNBC cells may exhibit aberrant nuclear morphology due to loss of appropriate chromatin organization or due to improper NE or nucleus formation in post-mitotic cells. TMPO expression has also been shown to inversely correlate with the level of nuclear membrane ruptures and this may play a role in the induction of abnormal nuclei (61).

TMPO expression has previously been shown to correlate with the proliferative capacity in multiple cellular models (62), supporting a role for TMPO in unregulated cancer cell proliferation. Unlike the other Lem-D proteins examined, depletion of TMPO was shown to induce significant anti-proliferative effects in both the TNBC and the non-cancerous MCF10A cells. Consistently, siRNA-mediated depletion of TMPO has previously been shown to induce growth-inhibiting phenotypes, such as reduced proliferation and the induction of apoptosis, in non-cancerous dermal fibroblasts and tumor models (63, 64). TMPO loss in Hutchinson Gilford progeria patient fibroblasts has also been casually linked to a proliferation defect in these cells (65). Previous investigations suggest that the role of TMPO in cell proliferation is likely due to TMPO’s role in pRb-mediated cell cycle control. TMPO can directly bind to Rb and is essential for the anchorage of Rb to the nucleus induces E2F activation and downstream gene expression and maintains appropriate cell cycle progression (66). TMPO has also been shown to have a key role in maintaining genomic stability, specifically via chaperoning replication protein A (RPA) to the site of DNA damage (67).

While the mechanism surrounding the lack of cancer specificity effect was not elucidated within our investigation, this does suggest that other Lem-D proteins may be more suited candidates as cancer therapeutics than TMPO, as there may be a heightened risk of toxicity to normal cells with this target.

### 4.5 Potential shared mechanisms of anti-cancer activity

Annexin V/PI apoptosis assays demonstrated that the extent of cell death did not fully correlate with the anti-proliferative effect induced by Lem-D protein depletion in TNBC cells. For instance, depletion of the Lem-D proteins was shown to reduce the proliferative capacity of MDA-MB-231 cells by >50%; however, only a 15%–20% decrease in viability was reported. Similar discrepancies were observed in BT549 cells following Lem-D protein depletion. It is possible that the anti-proliferative effect is not exclusively induced by cell death or occurs at a later point than the 5-day time point examined, and the true level of cell death is not being fully observed by our assay. Given the Lem-D proteins are known to have diverse functions, the proliferative arrest could be due to mitotic arrest or cellular senescence or quiescence after improper mitotic progression, which is feasible if targeting Lem-D proteins disrupts the reformation of the NE (37, 39, 68, 69). Therefore, future experiments should include investigating the role of these proteins in cell cycle progression, specifically mitosis.

It is evident that the Lem-D proteins have several unique functions in maintaining the NE structural integrity of interphase cells and during mitotic NE breakdown and reformation, with disruption of any of these processes uniquely leading to impaired NE integrity and a subsequent aberrant nuclear phenotype. However, it cannot currently be distinguished whether siRNA-mediated depletion of the individual Lem-D proteins may also induce aberrant nuclei in TNBC cells via a common mechanism mediated by their universal Lem-D, rather than their cellular functions discussed above.

To date, there have been minimal studies conducted in non-cancerous mammalian cells examining the effect of Lem-D protein depletion on the nuclear morphology of “normal” cells (70). Within our investigations, we have shown that, unlike TNBC cells, depletion of the Lem-D proteins does not significantly alter nuclear morphology in non-cancerous MCF10A cells. While we are unable to establish an exact mechanism by which depletion of the Lem-D proteins induces a tumor-specific outcome, several potential mechanisms should be investigated in further studies. It is well reported that nuclear morphology is frequently altered under endogenous conditions in tumor cells, with nuclear invaginations and folded nuclei being associated with higher malignancy rates and poor patient outcomes (71). Similarly, the criteria for pathological diagnosis of several cancers include the detection of aberrant NE protein expression or morphology (72).

While the localization of the Lem-D proteins is well established in non-diseased mammalian cells and other cancer models, it remains to be established whether this is maintained in TNBC cell lines (39, 49, 55, 73). We demonstrated that localization of Lem-D proteins to the INM is largely maintained in TNBC, and these proteins participate in cellular processes required to preserve TNBC nuclear morphology. While the underlying mechanism of these siRNA-induced aberrant nuclear morphologies was not within the scope of this investigation, several mechanisms have been considered based on known roles of the proteins.

Particularly, it is conceivable that tumor cells have a more prominent change in nuclear morphology following Lem-D protein depletion due to a mechanical vulnerability induced by their pre-existing abnormal nuclear structure, which promotes further distortion of the nucleus following disruption to NE homeostasis. Not only are the Lem-D proteins involved directly in maintaining nuclear morphology due to their NE localization and mitotic roles but they have also been identified as interactors of other proteins involved in maintaining nuclear stability, such as Lamin A/C and Lamin B (15, 73, 74). Expression of these proteins is also frequently mis-localized or decreased in breast cancer, thereby, suggesting that depletion of their Lem-D interactors may further impair the function of these proteins, emphasizing the reduction in nuclear stability induced by their dysregulation.

Our data demonstrate that siRNA-mediated depletion of Ankle2, Emerin, and Lemd2 inhibits cell proliferation in a largely tumor-specific manner in TNBC cells. Further investigations would benefit from the use of a xenograft model to validate the utility of targeting the Lem-D proteins in TNBC.

In conclusion, our works demonstrate the consistent localization of Ankle2, TMPO, Emerin, and Lemd2 to the NE in TNBC cells. siRNA-mediated depletion of these proteins also indicates a tumor-specific role of the Lem-D proteins in maintaining TNBC nuclear morphology. Furthermore, the induction of aberrant nuclei via depletion of these proteins produces an evident anti-proliferative effect and cell death in TNBC cells. Except for TMPO, this phenotype was demonstrated to be predominately tumor specific. Therefore, while further work is needed to elucidate the underlying mechanism by which the Lem-D proteins regulate TNBC growth, our findings provide the first evidence for a dynamic role of the Lem-D proteins in tumorigenesis and the potential for targeting this family as an anti-cancer therapy.

## Data availability statement

The original contributions presented in the study are included in the article/**Supplementary Material**. Further inquiries can be directed to the corresponding author.

## Ethics statement

Experimental procedures were approved by the Queensland University of Technology, Human Research Ethics Committee.

## Author contributions

EB conceived and directed the project. MR, CC, PS, and JB contributed to the laboratory experiments. All the authors contributed to project design and writing the manuscript. All authors contributed to the article and approved the submitted version.
