# Supplementary material for: The expression and role of the Lem-D proteins Ankle2, Emerin, Lemd2, and TMPO in triple-negative breast cancer cell growth
Source: Front Oncol. 2024 Apr 24;14:1222698. doi: 10.3389/fonc.2024.1222698 (PMC11076778; doi:10.3389/fonc.2024.1222698)
Supplement: Supplementary file 1 [file Image_1.pdf]

Supplementary Material

The Expression and Role of the Lem-D Proteins Ankle2, Emerin, Lemd2 and TMPO in Triple Negative Breast Cancer Cell Growth

Maddison Rose[1], Joshua T. Burgess[1], Chee Man Cheong[1], Mark N. Adams[1], Parastoo Shahrouzi[2], Kenneth J. O'Byrne[1,3], Derek J. Richard[1] and Emma Bolderson[1*]

*** Correspondence:** Emma Bolderson: emma.bolderson@qut.edu.au

1.1 Supplementary Figures

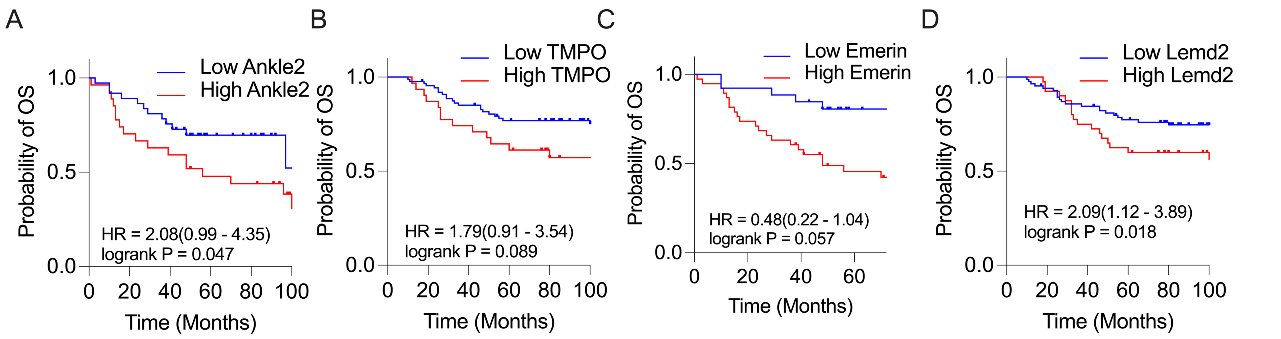

Supplementary Figure 1. Kaplan-Meier values for Lem-D protein expression in breast cancer patients showed high protein expression of Lem-D proteins was associated with a decrease in the probability of patient overall survival. Lem-D protein expression was categorised as high or low expressed based on the median protein expression within the database. The effect of: (a) Ankle2, (b) TMPO, (c) Emerin and (d) Lemd2 mRNA expression on patient overall survival. Statistical significance was calculated using a log-rank p-test.

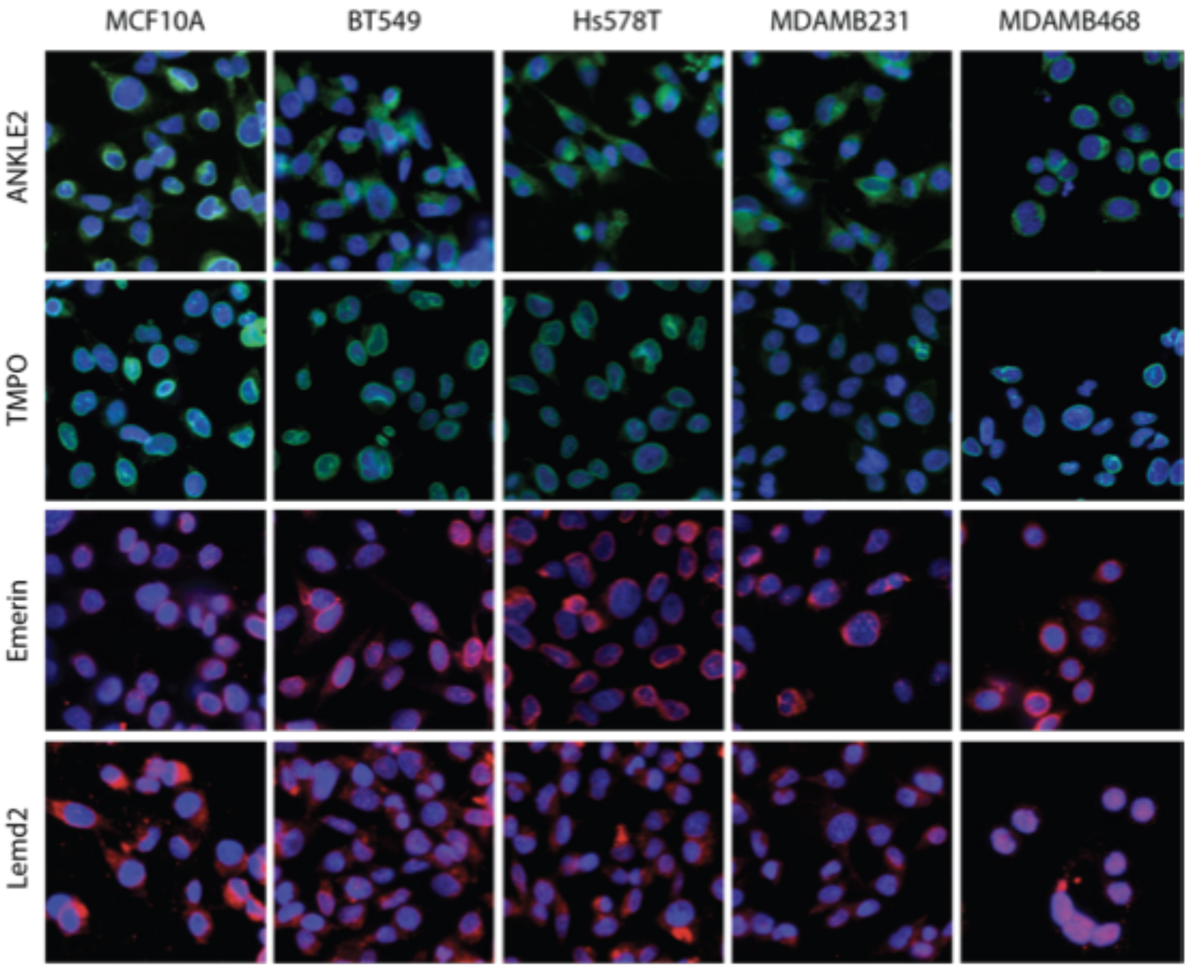

Supplementary Figure 2. Population view of MCF10A, BT549, HS578T, MDA-MB-231 and MDA-MB-468 cells showing localisation of Ankle2, TMPO, Emerin and Lemd2. Imaging was completed using the InCell Analyser 6500 (GE Science).

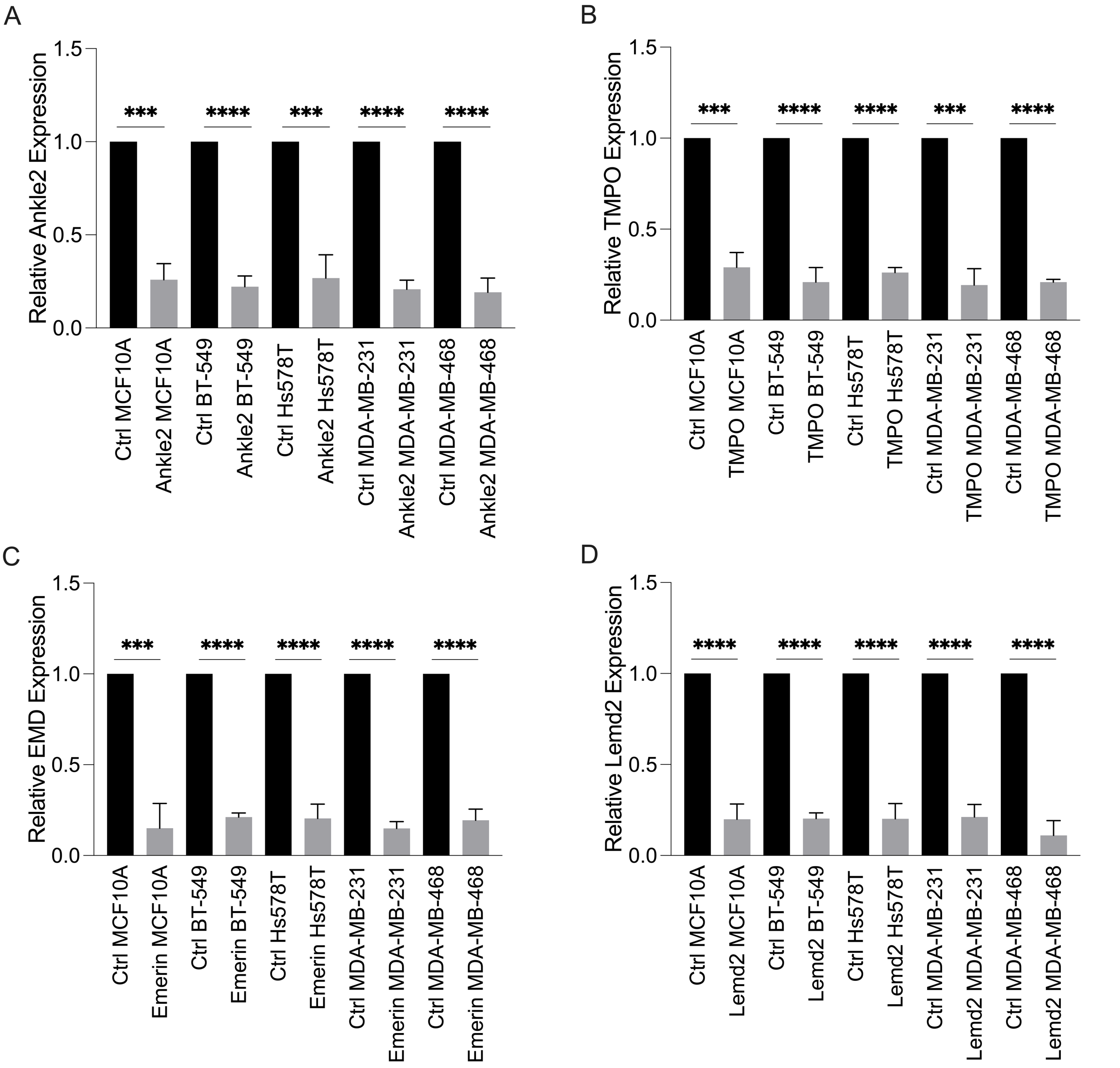

Supplementary Figure 3. Transfection of TNBC and MCF10A cells with the Lem-D siRNAs significantly decreases respective protein expression. Quantification of total cell expression in control and Lem-D siRNA transfected MCF10A, BT549, HS578T AND MDA-MB-231 cells 168 hours post-transfection was completed using the InCell Analyser 6500 (GE Science) and corresponding analysis software. (a) Ankle2 (b) TMPO (c) Emerin and (d) Lemd2 expression. Graphed values represent results from three individual repeats and error bars denote standard deviation of the mean. Statistical significance was calculated using an unpaired t-test: ****, < p 0.0001.

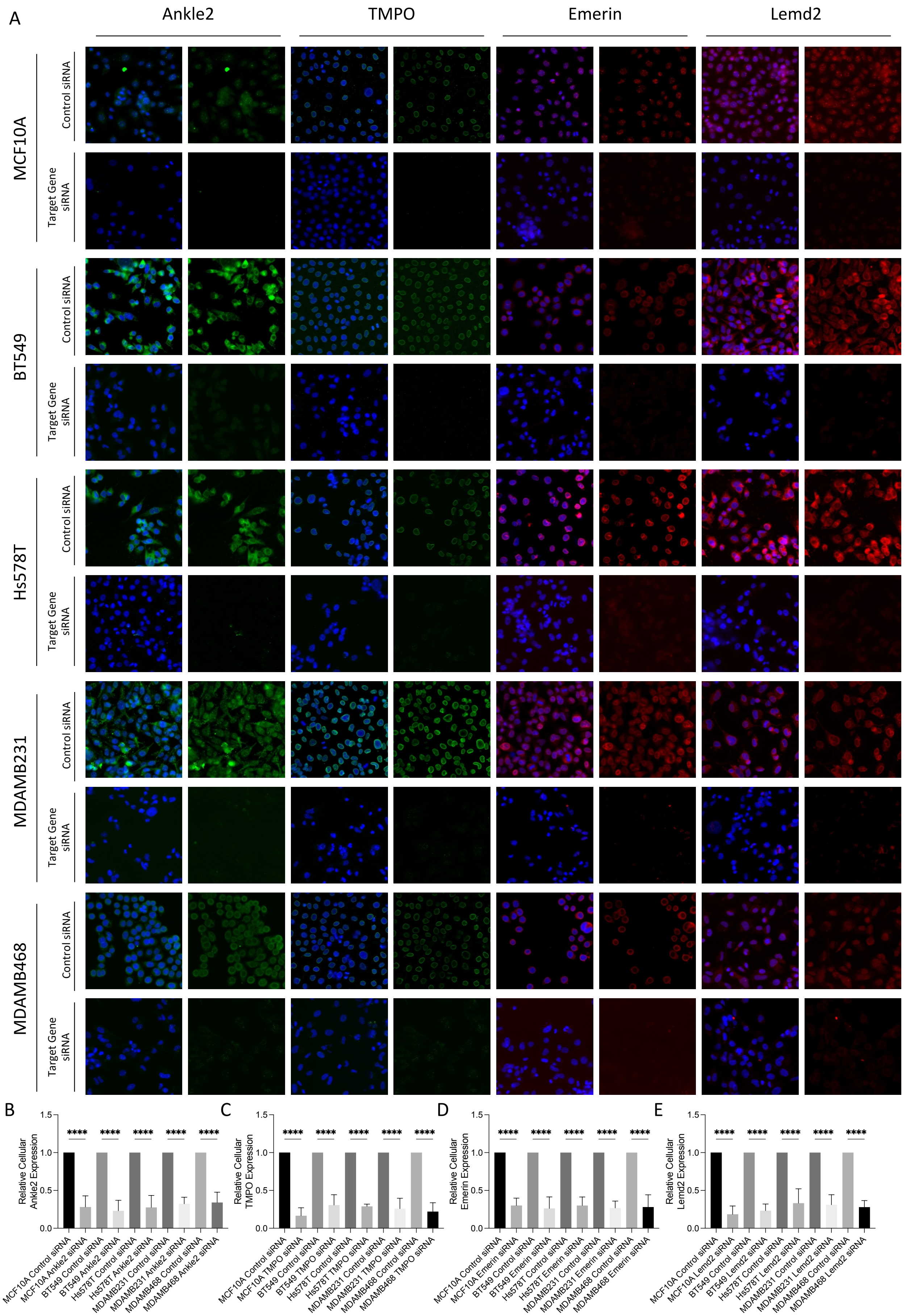
A
Ankle2
TMPO
Emerin
Lemd2
MCF10A
BT549
Hs578T
MDAMB231
MDAMB468
Control siRNA
Target Gene siRNA
B
C
D
E
Relative Cellular Ankle2 Expression
Relative Cellular TMPO Expression
Relative Cellular Emerin Expression
Relative Cellular Lemd2 Expression
****

Supplementary Figure 4. (a) Representative images demonstrating that transfection of MCF10A, BT549, HS578T AND MDA-MB-231 cells with the Lem-D siRNAs significantly decreases respective protein expression post siRNA transfection. Imaging was completed using the InCell Analyser 6500 (GE Science). Quantification of total cell expression in control and Lem-D siRNA transfected MCF10A, BT549, HS578T AND MDA-MB-231 cells was completed 72 hours post-transfection using the InCell Analyser 6500 (GE Science) and corresponding analysis software. (b) Ankle2 (c) TMPO (d) Emerin and (e) Lemd2 expression. Graphed values represent results from three individual repeats and error bars denote standard deviation of the mean. Statistical significance was calculated using an unpaired t-test: ****, < p 0.0001.

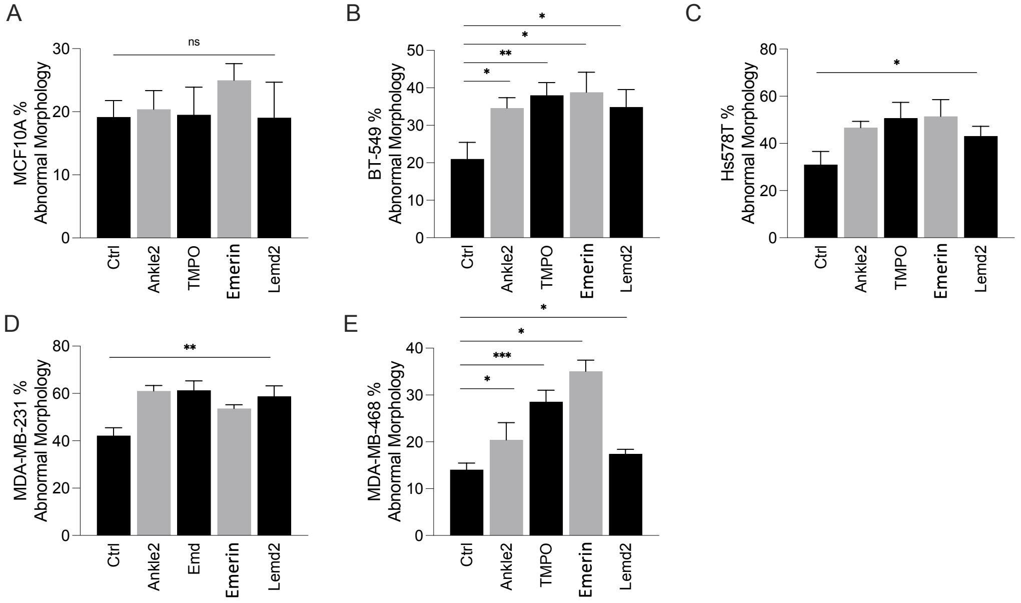

Supplementary Figure 5. Quantification of the proportion of cells where Ankle2, TMPO, Emerin and Lemd2 depletion via siRNA induced aberrant nuclear morphology in TNBC and MCF10A cells. Cells were visually quantified as having abnormal or normal nuclear morphology. (a) MCF10A (b) BT549 (c) Hs578T (d) MDA-MB-231 and (e) MDA-MB-468 cells transfected with Lem-D protein siRNAs (Ankle2, TMPO, Emerin and Lemd2) in comparison to their respective controls. Quantifications are based on 200 cells/condition in at least three independent experiments. Error bars denote standard deviation of the mean. Statistical significance was calculated using an unpaired t-test ***, < p 0.0002, **, $p < 0.0021$, *, $p < 0.0332$.

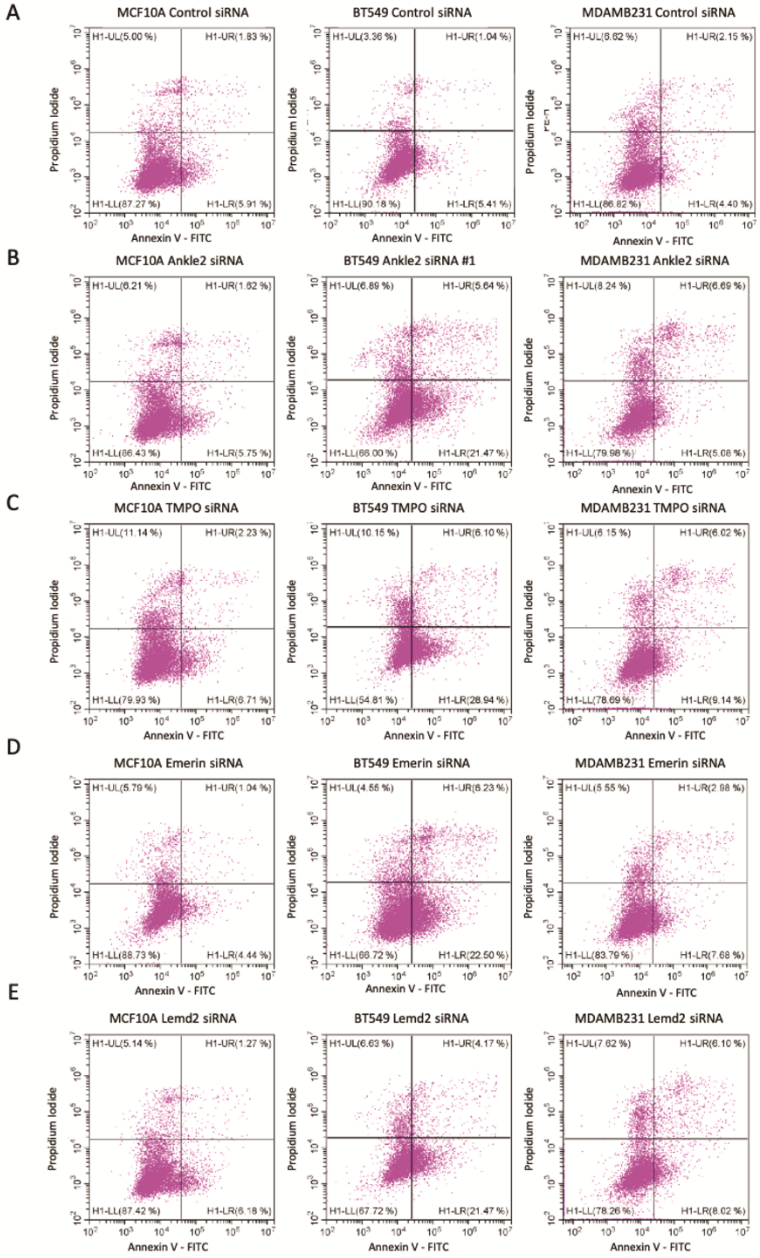

Supplementary Figure 6. Representative flow cytometry graphs for control and Lem-D protein siRNA transfected non-cancerous MCF10A cells and the representative TNBC cell lines, BT549 and MDA-MB-231. Live cells were stained with propidium iodide and Annexin V 488 and assayed using a CytoFLEX flow cytometer. (Q4): live cells, early– and late-apoptotic cells (Q3 and Q2) and necrotic cells (Q1). (a) Control siRNA, (b), Ankle2 siRNA (c) TMPO siRNA (d) Emerin siRNA and (e) Lemd2 siRNA.
